# Stigma and outness about sexual behaviors among cisgender men who have sex with men and transgender women in Eswatini: a latent class analysis

**DOI:** 10.1186/s12879-019-3711-2

**Published:** 2019-03-05

**Authors:** Carrie Lyons, Shauna Stahlman, Claire Holland, Sosthenes Ketende, Lynn Van Lith, Duncan Kochelani, Mpumelelo Mavimbela, Bhekie Sithole, Libet Maloney, Sibusiso Maziya, Stefan Baral

**Affiliations:** 1 Johns Hopkins Bloomberg School of Public Health, Department of Epidemiology, Baltimore Maryland, USA; 2 Johns Hopkins Center for Communication Programs (CCP), Baltimore, USA; 3 Johns Hopkins Center for Communication Programs (CCP), Mbabane, Swaziland; 4 Ministry of Health, Swaziland National AIDS Program, Mbabane, Swaziland; 5 Health Plus 4 Men, Manzini, Swaziland

**Keywords:** Stigma, Men who have sex with men, Transgender women, Disclosure, Swaziland

## Abstract

**Background:**

Men who have sex with men (MSM) and transgender women in Sub-Saharan Africa are subjected to high levels of sexual behavior-related stigma, which may affect mental health and sexual risk behaviors. MSM and transgender women who are open about, or have disclosed their sexual behaviors appear to be most affected by stigma. Characterizing the mechanism of action of stigma in potentiating HIV-risks among these key populations is important to support the development of interventions.

**Methods:**

In this study, a total of 532 individuals were recruited across Eswatini (Swaziland) through chain-referral-sampling from October – December 2014, including 419 cisgender MSM and 109 transgender women. Participants were surveyed about demographics, stigma, outness of same-sex practices to family members and healthcare workers, and mental and sexual health. This study used latent class analysis (LCA) to determine latent constructs of stigma/outness, and used multinomial logistic regression to determine associations with underlying constructs and sexual risk behaviors.

**Results:**

Three latent classes emerged: 1) Those who reported low probabilities of stigma (55%; 276/502); 2) Those who reported high probabilities of stigma including physical violence and fear/avoidance of healthcare, and were not “out” (11%; 54/502); and 3) Those who reported high probabilities of stigma including verbal harassment and stigma from family and friends, and were “out” (34%; 172/502). Relative to the “low stigma” class, participants from an urban area (adjusted odds ratio [AOR] = 2.78, 95% Confidence Interval [CI] = 1.53–5.07) and who engaged in condomless anal sex (AOR = 1.85, 95% CI = 1.17–2.91) were more likely to belong to the “high stigma, ‘out’” class. In contrast, those who had a concurrent male or female partner were more likely to belong to the “high stigma, not ‘out’” class AOR = 2.73, 95% CI = 1.05–7.07). Depression was associated with membership in both high-stigma classes (AOR = 3.14, 95% CI = 1.50–6.55 “not out”, AOR = 2.42, 95% CI = 1.51–3.87 “out”).

**Conclusions:**

Sexual behavior stigma at a community level is associated with individual-level risk behaviors among MSM and transgender women, and these associations vary by level of outness about sexual practices. Achieving sufficient coverage of evidence-based stigma interventions may be key to realizing the potential impact of HIV prevention and treatment interventions for MSM and transgender women in Eswatini.

## Background

The Kingdom of Eswatini, formerly Swaziland, has one of the world’s most widespread HIV epidemics, with more than 27% of adults aged 15–49 living with HIV in 2014 [1]. Encouragingly, in Eswatini and other countries with a generalized HIV epidemic, there has been a decrease in HIV incidence in recent years due to a coordinated response and increase in HIV prevention program coverage including antiretroviral therapy and prevention of mother-to-child transmission [2, 3]. However, the HIV prevalence among key populations including gay men and other men who have sex with men (MSM), as well as transgender women, is significant. In particular, HIV incidence among young MSM is increasing in almost every part of the world [4–6]. Subsequently, increasing effort is being dedicated to researching and addressing the HIV epidemic among these key populations even in the context of more broadly generalized epidemics [7, 8].

For cisgender MSM (cis-MSM) and transgender women, the potential effectiveness of HIV prevention and treatment programing may be limited by structural- and community-level factors, such as stigmas pertaining to sexual behaviors and gender identity, which contribute to suboptimal health-seeking behaviors [9, 10]. For example, culturally-insensitive health workers may result in cis-MSM and transgender women avoiding HIV prevention services, or cis-MSM and transgender women living with HIV may avoid HIV treatment services altogether. Reduced utilization of health and HIV services by cis-MSM and transgender women, due to enacted or perceived discrimination, may limit knowledge of the risks of condomless anal intercourse and opportunities for access to novel and emerging prevention services such as pre-exposure prophylaxis as it becomes increasingly available [11, 12]. Sexual behavior stigma may also increase risk for depression and other adverse mental health outcomes [13, 14]. In turn, adverse mental health outcomes may further increase risk for HIV by decreasing self-efficacy and increasing sexual risk behaviors including condomless anal sex with HIV status-unknown partners [15–17], and by affecting the desire or ability of cis-MSM and transgender women to engage in healthcare [18]. Sexual behavior stigma among these key populations may also limit stable couple formations resulting in larger sexual networks, in which people are less likely to know the HIV status of their sexual partners and may ultimately result in increased risk of HIV infection [19, 20].

Experienced sexual behavior stigma is often greater for cis-MSM and transgender women who have disclosed and are open about their identity or practices, even if these individuals are also more likely to be financially self-sufficient, comfortable about their sexuality, and have reduced minority stress after disclosure [20–23]. Potentially, this is because they are more easily identified as targets for discrimination or harassment by broader community members [22, 24]. However, non-disclosure of sexual behaviors can lead to poorer mental health, reduced engagement in HIV prevention services, and increased sexual risk-taking behaviors [25–27]. Thus, there is a paradox whereby coming out is associated with greater experiences of stigma even if it can result in improved mental health and HIV-related outcomes and greater awareness and acceptance of gay and transgender communities.

Among MSM in Eswatini, sexual orientation has been estimated to be three-fifths identifying as gay or homosexual, two-fifths as bisexual, and a small proportion reporting as heterosexual [28]. A study of transgender women and cis-MSM across 8 African countries showed Eswatini had a higher proportion of transgender participants than Malawi, Lesotho, Togo, and The Gambia [29]. There is a need to better understand the role of stigma in driving the persistent HIV epidemic among cis-MSM and transgender women in Eswatini. Especially considering the context of Eswatini with an estimated HIV prevalence of 13% among cis-MSM and transgender women [30], where same sex relations is a common law offence [31], and where stigma poses a potentially significant barrier to prevention programs and services.

The objectives of this study are: 1) to conduct a latent class analysis (LCA) to determine the latent constructs of stigma and disclosure status among cis-MSM and transgender women in Eswatini, and 2) to determine associations with underlying stigma constructs and sexual risk behaviors potentially putting these individuals at increased risk for HIV infection. We chose an LCA approach in order to explore how clusters of stigma and disclosure status were related to risk behaviors. LCA is a person-centered methodological approach to identify unobservable groups through patterns of responses across individuals. This approach aims to identify homogeneous groups that would be challenging to determine by assessing indicators individually [32]. Stigma attributable to sexual behavior is driven through social processes, and may manifest through multidirectional, and mutually reinforcing mechanisms [33]. Therefore, utilizing a person-centered latent approach to assess sexual stigma, outness, depression, sexual risk behaviors, and sociodemographics help to better understand these complex patterns. By capturing the multiplicity of the stigma/outness items, the objective was to better understand how these items can be conceptualized and captured in relation to sexual risk behavior among these individuals.

## Methods

### Study population and design

A total of 532 individuals were recruited across 5 cities/towns and surrounding regions (Lavumisa, Manzini/Matsapha, Mbabane/Ezulwini, Nhlangano, and Piggs Peak) in Eswatini through peer-referral sampling from October – December 2014. In order to be eligible for the study, participants had to report being assigned the male sex at birth, being aged 18 years or older, having insertive and/or receptive anal sex with a man within the past 12 months, speaking siSwati or English, and being capable of providing written informed consent. This study was approved by the Johns Hopkins Bloomberg School of Public Health Institutional Review Board and the Eswatini Scientific and Ethics Committee.

### Data collection and key measures

During the study visit, trained interviewers administered a structured questionnaire through a face-to-face interview in a private location. The questionnaire included questions about demographics, stigma, disclosure about having sex with men, and mental and sexual health.

### Demographics

A two-step gender assessment was used to distinguish between cis-MSM and transgender women in this study. This assessment included reported sex at birth, and reported current gender identity [34, 35]. Individuals who reported a gender identity as female or intersex were considered transgender women in these analyses. Participants who reported a gender identity of male are defined as cis-MSM. For these analyses, we included information on age, highest level of completed education, gender identity, employment status (employed or not employed), and whether the study site was located in an urban or peri-urban area. In order to perform the LCA, each of these variables was dichotomized into binary indicators.

### Sexual behavior stigma

Stigma attributable to having sex with men was measured by asking a series of “yes” or “no” questions, which have been used in several previous studies of cis-MSM and transgender women in Sub-Saharan Africa [10, 36]. This sexual behavior stigma was comprised of stigma from personal, social, and healthcare settings. Personal-life stigma included feeling excluded at family gatherings, feeling that family members made discriminatory remarks or gossiped, or feeling rejected by friends. Social stigma included feeling that the police refused to protect you, feeling scared to walk around in public places, being verbally harassed, blackmailed, physically hurt, or tortured, as well as experience of violence. Finally, healthcare stigma included feeling that you were not treated well in a healthcare center, hearing healthcare providers gossip, feeling afraid to go to healthcare services, or avoiding healthcare services.

### “Out” about having sex with men

Participants were asked, “Have you told any member of your family that you have sex with men or that you are attracted to other men?” as well as, “Does anyone in your family know that you have sex with other men or that you are attracted to other men, other than those who you have told?” Participants who reported “yes” to either were considered being “out” to family members. Participants who responded “yes” to the question, “Was there a time when any health care provider learned that you have sex with other men or that you are attracted to other men (for example, you told them, or they found out because someone else told them)?” were considered being “out” to heath care workers.

### Depression

A positive depression screen was defined as a Patient Health Questionnaire (PHQ-9) score of 10 or greater [37]. The PHQ-9 measures the frequency of depression symptoms within the past two weeks. This scale has been used previously in Sub-Saharan African populations [38, 39] and had good internal consistency in our study sample (Cronbach’s alpha = 0.89).

### Sexual risk practices

Participants were asked how often condoms were used within the past 12 months for receptive and insertive anal sex. These measures were dichotomized into a single indicator for condomless anal sex that included “any” or “none”. In addition, participants were asked if there was any time in the last 12 months that they had multiple regular sexual partnerships at the same time; that is involved in two or more ongoing sexual partnerships, either with males or female partners. These measures were dichotomized into a single indicator for concurrent sexual partnerships that included “any” or “none”.

### Statistical analyses

We tabulated descriptive characteristics of participants using frequencies and percentages. Bivariate logistic regression was used to test associations between being “out” about having sex with men and sexual behavior stigma. These analyses were conducted using SAS software Version 9.4 (Cary, NC, USA).

In a two-step process, we first used LCA to identify classes based on self-reported measures of stigma, and whether or not it was known to family or healthcare workers that the participant had sex with men. Two- through six- latent class models were produced iteratively. The number of classes was selected based on theoretical and practically meaningful patterns as well as model fit criteria (i.e., goodness-of-fit indices). Fit indices included the likelihood ratio test statistic (G^2^), the Akaike information criterion (AIC), the Bayesian information criterion (BIC), the consistent AIC (CAIC), and entropy (Table 1) [40]. Smaller values of AIC and BIC and higher values of entropy indicate better fit.

**Table 1:** Goodness-of-Fit Indices Comparing Class Models of Stigma and Being “Out” among MSM and Transgender Women in Eswatini, 2014

Class	G^2^ (df)	AIC	BIC	CAIC	Entropy
2	2608 (32736)	2670	2803	2833	0.87
3	2155 (32720)	2249	2450	2497	0.90
4	1890 (32704)	2016	2285	2348	0.90
5	1757 (32688)	1915	2253	2332	0.88
6	1680 (32672)	1870	2277	2372	0.90

*AIC* Akaike information criterion, *BIC* Bayesian information criterion, *CAIC* Consistent Akaike information criterion, *G*^2^ Likelihood ratio test statistic

Next, multinomial logistic regression was used to identify demographic characteristics, sexual risk behaviors, and mental health characteristics (i.e., depression) that were associated with class membership. These variables were first analyzed individually and then simultaneously in a multivariable model. All covariates except for age and reporting more than a high school education were found to be significant predictors of membership in at least one latent class in the bivariate analyses (not shown). Demographic variables considered to have theoretical importance were kept in the final model regardless of their level of statistical significance. As a result, no variables were dropped from the final model. For both the LCA and logistic regression, participants with missing data were excluded (*N* = 30). Less than 1% of data were missing for all variables in the LCA and fewer than 4% were missing for variables in the logistic regression. The two-step process analyses were performed using SAS PROC LCA [41, 42].

## Results

### Participant characteristics

Prevalence of participant characteristics is presented in Table 2. A total of 532 individuals participated in this study, including 419 (79.4%) cis-MSM and 109 (20.6%) transgender women. Participants ranged in age from 18 to 50 years, with a median age of 24 years and an interquartile range of 22–28 years. Less than one-quarter (*n* = 113, 21.2%) had completed secondary school or less, whereas 51.1% (*n* = 272) had completed high school and 27.6% (*n* = 147) completed more than a high school education. The majority of participants were sampled from an urban study site (*n* = 400, 75.2%) and a little more than one-half were employed or students (*n* = 301, 56.6%). Experiences of stigma ranged in prevalence from 10.9–43.7% depending on the type of stigma. Almost 44% (*n* = 233) were out to family members whereas 20.5% (*n* = 108) were out to healthcare providers.

**Table 2:** Characteristics of MSM and Transgender Women Participants, Eswatini 2014 (*N* = 532)

*Characteristics*	Total	
	n	%
Age, in years		
< =24	280	52.6
> 24	252	47.4
Gender identity		
Man	419	79.4
Woman/Intersex	109	20.6
Education completed		
Secondary school or less	113	21.2
High school	272	51.1
More than high school	147	27.6
Employed	301	56.6
Study site location		
Urban	400	75.2
Peri-urban	132	24.8
Depression	201	39.1
Openess about sexual behavior		
Told any family member or any family member knows that individual has sex with men	233	43.8
Told any healthcare provider or any healthcare provider knows that individual has sex with men	108	20.5

### Associations between sexual behavior stigma and being “out”

Being out to a family member was associated with feeling excluded by family members (Odds Ratio [OR] = 2.01, 95% Confidence Interval [CI] = 1.35, 3.00), feeling gossiped about by family members (OR = 4.07, 95% CI = 2.77, 5.98), feeling rejected by friends (OR = 4.44, 95% CI = 2.83, 6.97), feeling like police refused to protect (OR = 1.78, 95% CI = 1.09, 2.89), feeling scared to walk around in public places (OR = 1.61, 95% CI = 1.13, 2.29), being verbally harassed (OR = 4.21, 95% CI = 2.92, 6.06), and being blackmailed (OR = 2.51, 95% CI = 1.65, 3.83). It was not significantly associated with being physically hurt (OR = 1.24, 95% CI = 0.81, 1.91), being tortured (OR = 0.93, 95% CI = 0.59, 1.45), being treated poorly in a healthcare setting (OR = 0.71, 95% CI = 0.40, 1.25), being gossiped about by a healthcare worker (OR = 1.22, 95% CI = 0.74, 2.00), being afraid to seek healthcare services (OR = 0.87, 95% CI = 0.61, 1.24), or avoiding seeking healthcare services (OR = 0.97, 95% CI = 0.68, 1.39) (Table 3).

**Table 3:** Stigma and Outness among MSM and Transgender Women Participants, Eswatini 2014 (N = 532)

*Stigma*	Total		Out to Family		Out to Healthcare provider	
	n	%	OR	95% CI	OR	95% CI
Personal-life stigma as a result of having sex with men						
Felt excluded at family gatherings	130	24.5	2.01	1.35, 3.00	1.64	1.03, 2.60
Felt that family members made discriminatory remarks or gossiped	173	32.6	4.07	2.77, 5.98	2.50	1.62, 3.87
Felt rejected by friends	116	21.9	4.44	2.83, 6.97	3.91	2.47, 6.19
Social stigma/violence as a result of having sex with men						
Felt police refused to protect you	77	14.6	1.78	1.09, 2.89	1.68	0.97, 2.91
Felt scared to walk around in public places	200	37.7	1.61	1.13, 2.29	1.47	0.96, 2.26
Verbally harassed	232	43.7	4.21	2.92, 6.06	3.63	2.31, 5.71
Blackmailed	117	22.0	2.51	1.65, 3.83	2.66	1.67, 4.22
Physically hurt	104	19.6	2.51	0.81, 1.91	1.49	0.90, 2.45
Tortured	95	18.0	0.93	0.59, 1.45	1.30	0.77, 2.19
Healthcare stigma as a result of having sex with men						
Felt not treated well in a health center	58	10.9	0.71	0.40, 1.25	2.49	1.39, 4.46
Heard healthcare providers gossiping	73	13.8	1.22	0.74, 2.00	2.16	1.25, 3.71
Felt afraid to go to healthcare services	193	36.4	0.87	0.61, 1.24	1.37	0.89, 2.11
Avoided going to healthcare services	185	34.8	0.97	0.68, 1.39	1.81	1.18, 2.79

Being out to a healthcare worker was associated with being treated poorly in a healthcare setting (OR = 2.49, 95% CI = 1.39, 4.46), being gossiped about by a healthcare worker (OR = 2.16, 95% CI = 1.25, 3.71), avoiding seeking healthcare services (OR = 1.81, 95% CI = 1.18, 2.79), feeling excluded by family members (OR = 1.64, 95% CI = 1.03, 2.60), feeling like family members gossiped (OR = 2.50, 95% CI = 1.62, 3.87), feeling rejected by friends (OR = 3.91, 95% CI = 2.47, 6.19), being verbally harassed (OR = 3.63, 95% CI = 2.31, 5.71), and being blackmailed (OR = 2.66, 95% CI = 1.67, 4.22). It was not significantly associated with feeling like police refused to protect (OR = 1.68, 95% CI = 0.97, 2.91), feeling scared to walk around in public places (OR = 1.47, 95% CI = 0.96, 2.26), being physically hurt (OR = 1.49, 95% CI = 0.90, 2.45), being tortured (OR = 1.30, 95% CI = 0.77, 2.19), or being afraid to seek healthcare services (OR = 1.37, 95% CI = 0.89, 2.11).

### Latent class analysis

#### Identification of latent classes

AIC, BIC, and CAIC values began to level-off at 3 latent classes and were primarily leveled-off at 4 classes. Purely based on model fit indices, a 4-class model might have been selected. However, after comparing conditional probability distributions between the 3-class and 4-class models, a 3-class model was selected based on the existence of meaningful risk profiles for participants [40, 42–44]. In brief, for the 4-class model, the high risk “not out” class appeared to divide into two groups: both had high levels of family gossip and verbal harassment whereas one group had higher levels of perceived healthcare stigma. We considered these to be sub-groups of the high risk “not out” class and maintained the 3-class model for ease of interpretation.

The first class (55%; 276/502) consisted of cis-MSM and transgender women who demonstrated overall low probabilities of stigma as a result of having sex with men (“low stigma” class) (Table 4). The conditional probability of being out to family members and healthcare workers was 38% and 15%, respectively, which suggests that some of the participants in this class were out to family members and healthcare workers although it was not a defining feature of this class. Individuals in the second class (11%; 54/502) exhibited high probabilities (> 0.50) of physical violence, torture, and fear/avoidance of seeking healthcare, and were less likely to have their sexual identities known by family members or healthcare workers (“high stigma, not ‘out’” class). Finally, the third class (34%; 172/502) demonstrated high probabilities of being excluded by or gossiped about by family members, verbal harassment, feeling scared to walk around in public, fear/avoidance of healthcare workers, and were more likely to have their sexual identities known by family members or healthcare workers (“high stigma, ‘out’” class).

**Table 4:** Sample Prevalence, Latent Class Probability, and Conditional Probability of the Final 3-Class Model for the Analytic Sample (*N* = 502), Eswatini 2014

	Total N (%)	Low stigma	High stigma, not “out”	High stigma, “out”
Unconditional probability of each class		0.54	0.11	0.34
Conditional probability of endorsing each item				
Personal-life stigma as a result of having sex with men				
Felt excluded at family gatherings	125 (25)	0.06	0.02	0.62
Felt that family members made discriminatory remarks or gossiped	165 (33)	0.16	0.00	0.71
Felt rejected by friends	109 (22)	0.09	0.00	0.48
Social stigma/violence as a result of having sex with men				
Felt police refused to protect you	71 (14)	0.05	0.15	0.28
Felt scared to walk around in public places	194 (39)	0.24	0.01	0.73
Verbally harassed	220 (44)	0.25	0.00	0.87
Blackmailed	110 (22)	0.12	0.04	0.44
Physically hurt	96 (19)	0.02	0.62	0.33
Tortured	87 (17)	0.03	0.67	0.25
Healthcare stigma as a result of having sex with men				
Felt not treated well in a health center	53 (11)	0.00	0.51	0.15
Heard healthcare providers gossiping	70 (14)	0.02	0.55	0.21
Felt afraid to go to healthcare services	187 (37)	0.07	0.85	0.72
Avoided going to healthcare services	178 (36)	0.05	0.79	0.72
Openness about sexual behaviors				
Told any family member or any family member knows he has sex with men	218 (43)	0.38	0.11	0.63
Told any healthcare provider or any healthcare provider knows he has sex with men	102 (20)	0.15	0.05	0.34

#### Relationships with class membership

In the final adjusted multinomial model, depression was associated with both high stigma classes relative to the low stigma class (*P* < 0.01) (Table 5). Reporting concurrent sex partners (*P* < 0.01) was associated with membership in the high stigma not out class whereas condomless anal sex was associated with membership in the high stigma out class (*P* < 0.01). Being employed and identifying with female/other gender was associated with reduced likelihood of membership in the high stigma not out class relative to the low stigma class (*P* < 0.05 and *P* < 0.05, respectively). Completing high school and more than a high school education were both associated with membership in the high stigma not out class relative to the low stigma class (*P* < 0.01 and *P* < 0.05, respectively). Being sampled from an urban area study site was associated with membership in the high stigma out class (*P* < 0.01). Age was not associated with class membership (*P* = 0.86).

**Table 5:** Multivariable Relationships with Latent Class Membership (N = 502), Eswatini 2014

	Latent Class							
	High stigma, not out (54)			High stigma, out (172)			Low stigma (276)	
Variable	N (%)	aOR	95% CI	N (%)	aOR	95% CI	N (%)	Ref.
Age (> 24 years)	30 (11.2)	1.07	0.53, 2.16	92 (34.5)	0.90	0.58, 1.41	145 (54.3)	1
Urban	42 (11.3)	1.93	0.84, 4.41	150 (40.3)	2.78	1.53, 5.07**	180 (48.4)	1
Transgender	2 (1.9)	0.12	0.02, 0.78*	49 (46.7)	1.21	0.72, 2.04	54 (51.4)	1
High school education	38 (14.6)	6.81	1.87, 24.82**	85 (32.7)	1.10	0.62, 1.93	137 (52.3)	1
More than high school education	13 (9.6)	5.27	1.22, 22.72*	50 (37.0)	1.16	0.59, 2.26	72 (53.3)	1
Employed	20 (7.1)	0.42	0.20, 0.90*	101 (35.8)	1.12	0.70, 1.81	161 (57.1)	1
Condomless anal sex	6 (3.6)	0.74	0.33, 1.70	71 (43.0)	1.85	1.17, 2.91**	88 (53.3)	1
Concurrent sex partners	5 (5.5)	2.73	1.05, 7.07**	63 (69.2)	0.93	0.58, 1.48	23 (25.3)	1
Depressed	33 (17.3)	3.14	1.50, 6.55**	87 (45.6)	2.42	1.51, 3.87**	71 (37.2)	1

*AOR* Adjusted Odds Ratio, *CI* Confidence Interval

*P < 0.05; **P < 0.01

## Discussion

Sexual behavior stigma is affecting cis-MSM and transgender women across Sub-Saharan Africa [13, 45–47], and is likely exacerbated by the illegality of same sex practices with punishments including fines or imprisonment [48]. Stigma and discrimination towards cis-MSM and transgender women have previously been associated with poor HIV-related health outcomes including reduced rates of HIV testing, increased risk for HIV infection, lower likelihood of discussing or disclosing HIV/AIDS status with male partners, and engagement in HIV treatment for those living with HIV, and increased condomless anal sex [49–52]. In these analyses, we found that outness about sexual behaviors grouped together with increased burden of multiple forms of stigma, and that these latent stigma/outness classes were associated with different types of sexual risk behaviors.

In Eswatini, there is persistent societal discrimination against the LGBT community backed by colonial-era legislation that prohibits anal sex between men [53]. As a result, LGBT individuals risk the loss of family members, friends, and employment if they disclose or are out about their sexual behaviors or gender identity. This structural-level stigma is manifested at the individual-level in our study. For example, participants who reported that family members knew about their sexual behaviors greatly increased the odds of reporting feeling excluded and gossiped about by family members. Similarly, having healthcare workers who knew about one’s sexual behaviors increased the odds of reporting poor treatment from healthcare workers, being gossiped about by healthcare workers, and avoiding seeking healthcare services. This is additionally problematic because disclosure of sexual practices to healthcare workers is necessary for obtaining accurate sexual histories and meaningful assessments of HIV risk, but in reality, disclosure can be very challenging. In the context of HIV prevention and treatment strategies in Eswatini, if cis-MSM and transgender women face stigma for disclosing their sexual practices, they may be less likely to disclose and subsequently less likely to be identified as appropriate candidates for novel biomedical HIV prevention services including pre-exposure prophylaxis.

In the latent class regression, those with concurrent male or female sexual partners were more likely to belong to the high stigma not out class. This finding is consistent with results from recent qualitative work examining intersecting stigmas among MSM in Eswatini, where participants reported that the secretive nature of MSM relationships led to greater numbers of sexual partners and more casual types of partners in some cases [19]. Participants indicated that because their MSM relationships are kept secret, families do not play a role in relationship counseling and peacekeeping in the same way that they might for heterosexual couples. It is also common for MSM in Eswatini and other regions to have girlfriends or wives, potentially to fulfill cultural expectations, further challenging the formation of stable male couples [19, 20]. In other settings, MSM who also have sex with women showed a higher risk of experiencing intimate partner violence, including physical violence and being threatened with disclosure of sexual orientation, than MSM with only male partners [54]. This may provide insight on the high probability of experienced violence among the high stigma, not out class in this study.

Prevention science theoreticians and practitioners have called for combination HIV prevention strategies, which would integrate a package of biomedical, behavioral, and structural interventions to address multiple layers of HIV risk [55–59]. These combination tactics are likely even more efficient for high risk MSM and transgender women in reducing HIV incidence [60–62]. But given the increased instances of condomless anal sex among those in the high stigma out group in this study, this suggests that structural interventions to address stigma will also be needed to reduce HIV risk behaviors; such as sensitivity training for healthcare workers and political advocacy to reduce or mitigate the effects of stigma. In Eswatini, the implementation and optimization of combination approaches are currently challenged by punitive policies and stigma affecting MSM [55, 57].

Those who identified with a non-male gender (including female or intersex) were least likely to belong to the high stigma and not out class. They were more likely to belong to the high stigma and out class although this was not found to be statistically significant. Previous work indicates that transgender women, or individuals assigned male sex at birth but who identify as a woman, are more likely to experience high levels of stigma in comparison even to MSM [29, 63, 64]. Thus, our findings may reflect the notion that transgender women are more likely to be visible in the community as compared to MSM who follow more traditional gender norms, and thus may be more easily targeted for stigma, discrimination, and other forms of abuse. Living in an urban residence being associated with belonging to the high stigma out class was not surprising and likely reflects patterns seen in the US and other high income settings where gay men and other MSM move to larger cities for social networking opportunities and a more tolerant social climate [65, 66].

Screening positive for depression on the PHQ-9 was associated with membership in each of the high stigma classes, compared to the low stigma class. This is consistent with previous data suggesting that depression is higher among MSM as compared with heterosexual men in many parts of the world potentially as a result of stigma and minority stress [13, 67–70]. MSM interviewed for a qualitative study in Eswatini indicated that living with a stigmatized identity led to feelings of depression and self-stigma [19]. Our findings here further highlight the strong and consistent impact that stigma appears to have on mental health, regardless of whether one is open about their sexual behavior. Unfortunately, there is virtually no literature describing effective depression interventions for MSM in Sub-Saharan Africa [71–73].

The latent class, low stigma, showed moderately high levels of disclosure to family and healthcare providers, however was not a defining characteristic of the class. The context of overall low stigma may provide for a supportive environment for disclosure of sexual behaviors. Although, the low stigma class still showed moderate levels of fear to be in public spaces and verbal harassment, and a higher conditional probability for these stigma measures than those in the high stigma, not out.

Potential limitations to our study include the use of cross sectional data, impeding the inference of causal relationships, and the non-random selection of study participants, which is an assumption of LCA. However, “hidden” populations such as cis-MSM and transgender women are difficult to sample through traditional methods given the lack of sampling frame including census-level data in Eswatini and peer-driven sampling approaches are more appropriate. Social desirability bias may have affected participant responses; for example, by causing underreporting of condomless anal sex and stigmatizing experiences. Although LCA leaves open the possibility that one or a few particular stigma items may be driving the associations with risk behaviors, we opted to use LCA to explore how clusters of stigma/outness were related to risk behaviors. The stigma metrics used in this study were self-reported stigma measures defined as attributable to sexual behavior. However, for individuals experiencing layered or intersecting stigma, the attributable characteristic of stigma may be difficult to identify. An additional limitation is that this sample was underpowered to conduct a separate analysis for transgender women without cis-MSM.

## Conclusion

Even in the context of increasingly available biomedical HIV intervention strategies including oral pre-exposure prophylaxis, the reduction of HIV-related risk practices remains crucial for the prevention of HIV acquisition and transmission. In these analyses, stigma appears to consistently be associated with increased HIV-related risk practices and risks for depression. Consequently, evidence-based stigma interventions that are able to operate under challenging legal and human rights settings may be key to combating the persistent HIV epidemic for cis-MSM and transgender women in Eswatini.

## Abbreviations

AIC: Akaike information criterion
AOR: Adjusted Odds Ratio
BIC: Bayesian information criterion
CAIC: Consistent Akaike information criterion
CI: Confidence Interval
HIV: human immunodeficiency virus
LCA: latent class analysis
MSM: men who have sex with men
PHQ: Patient Health Questionnaire
